# Evaluation of machine learning models for classification of fertilization status of African clawed frog (*xenopus laevis*) by use of synthetic images

**DOI:** 10.1371/journal.pone.0360384

**Published:** 2026-11-11

**Authors:** Gopal Narayan Srivastava, Monika Pandey, Kiran Bist, Sandesh Chapagain, Carl Anderson, Nikko-Ideen Shaidani, Marko E. Horb, Peter Wolenski, Terrence R. Tiersch, Yue Liu

**Affiliations:** 1 Aquatic Germplasm and Genetic Resources Centers, Louisiana State University Agricultural Center, Louisiana, United States of America; 2 Department of Biological Sciences, Louisiana State University, Baton Rouge, Louisiana, United States of America; 3 Department of Mathematics, Louisiana State University, Baton Rouge, Louisiana, United States of America; 4 Department of Computer Science, Louisiana State University, Baton Rouge, Louisiana, United States of America; 5 National Xenopus Resource (NXR), Marine Biological Laboratory, Massachusetts, United States of America; 6 National Aquatic Germplasm Center (NAGC), Louisiana State University Agricultural Center, Louisiana, United States of America; Fukushima Medical University, JAPAN

## Abstract

The African clawed frog (*Xenopus laevis*) has been a pivotal model organism providing valuable insights into cell biology, developmental biology, and human disease. Fast and accurate identification of fertilized embryos versus unfertilized *X. laevis* eggs is crucial for time sensitive experimental applications such as assessing the efficacy of sperm cryopreservation and artificial fertilization. Traditionally, this task has relied on manual annotation, a labor-intensive process. To address this challenge, we developed a convolutional neural network (CNN)-based tool using the *YOLOv10b* (“You only look once version 10 balanced accuracy”) architecture to detect and quantify fertilized and unfertilized eggs in images. Our tool leveraged synthetic data generation to automate the creation of 1,194 training and validation images and annotations, significantly reducing the need for manual annotation. Through testing on 120 synthetic and 20 experimental images, we evaluated the performance of three optimizer models: Adam, AdamW, and SGD. The accuracy when testing the synthetic data was 87% for the Adam model, 89% for the AdamW model, and 90% for the SGD. On the other hand, the median percentage embryo classification accuracy (ECA) values were 97% (Adam), 95% (AdamW), and 96% (SGD). Our results demonstrate the feasibility of rapid development of machine learning tools for classification of fertilization making it a valuable tool for improving the efficiency in laboratory operation for biomedical researchers and resource centers that use *X. laevis* as a model organism. The codes to image synthesizing, training, and testing along with the Graphical User Interface (GUI) were made publicly available on GitHub and Open Science Framework (OSF).

## Introduction

The advancement of biomedical research has long relied on centralized animal resource centers which provide standardized, high-quality biological materials essential for reproducible experimentation. These centers ensure consistent access to validated animal models, preserve genetic lines, and streamline the dissemination of crucial biological and genetic resources across the scientific community. Among the widely utilized aquatic organisms maintained in such centers, the National *Xenopus* Resource (NXR) [1] at the Marine Biological Laboratory, Woods Hole, MA, holds a pivotal role. The NXR supplies healthy, genetically characterized *Xenopus laevis*, *Xenopus tropicalis*, and several other related species to researchers worldwide, and plays a central role in training, community coordination, and technological innovation for *Xenopus*-based studies.

*Xenopus* clawed frogs have been a cornerstone of biological research, alongside mouse (*Mus musculus*), zebrafish (*Danio rerio*), nematode (*Caenorhabditis elegans*), and fruit fly (*Drosophila melanogaster*) [2]. For *Xenopus* this is primarily due to their large egg clutches, ease of egg and embryo manipulation, external embryonic development, and development analogous to humans. The scientific investigation of *Xenopus laevis* has significantly advanced our understanding of gene regulation, cell-cycle control, and signal transduction, among other areas [3]. Aquatic stock centers have recently begun to increase operational efficiency and protect genetic resources by using cryopreservation to support development of germplasm repositories [4,5]. This research and its application rely on quick and accurate identification of *in vitro* fertilization of eggs to develop, improve and use protocols [6].

The practical advantages of *X. laevis*—including its capacity to lay thousands of eggs in a single clutch—present a logistical challenge: the need to efficiently and accurately identify fertilization status, especially for researchers who rely on developmental timing to assess the effects of gene manipulation, pharmacological interventions, or environmental exposures. While manual characterization of embryos has historically been the norm, it is time-consuming, subjective, and impractical for large-scale experiments [3]. Traditionally, the identification and enumeration of *X. laevis* embryo stages have been performed manually, a labor-intensive process and identifying fertilization would be a first step in monitoring development. Researchers have commonly used the ImageJ tool [7] within the Fiji application [8] to count eggs from images, but this method also demands significant time and effort. In addition, identification of development stages by use of the naked eyes can lead to issues in reproducibility in the results and training.

Therefore, to support a long-term goal of developing a comprehensive computational tool capable of accurately identifying and counting embryos at various developmental stages, it is essential to enable characterization of fertilized and unfertilized eggs as a feasibility study. In addition, to speed the model training process we recognized the need for an approach to creation of a synthetic data set. As such, the overall goal of this work was to develop a convolutional neural network (CNN)-based tool that can classify and quantify images of fertilized and unfertilized eggs of *X. laevis* in a Petri dish [9]. This tool employed the *YOLOv10b* [10] architecture for object detection and yielded high accuracy and precision in determining fertilization status. The specific objectives were to: 1. create an algorithm to generate a synthetic image dataset with fertilized and unfertilized *X. laevis* embryos; 2. train convolutional neural network models for the classification of fertilized and unfertilized embryos, and 3. create graphical user interface for easy access of the tool by users including experimental biologists and conservationists.

Multiple tools have been published previously that utilize CNN architecture to detect development stages in research models such as zebrafish (*Danio rerio*) [11]. To our knowledge, this is the first report of classifying eggs from *Xenopus* frogs by use of machine learning models. In addition, this work is innovative in eliminating the manual annotation process for labeling of training images. The synthetic images and YOLO annotation process were automated for generating effective training data. This approach eliminated the bottleneck of manual dataset marking, enabling efficient training of object detection models on a large, restricted dataset. We also developed GUI to be used in the field by scientists and researchers which can classify eggs in more than 20 images within 5 minutes. The development of this work involved a collaborative team spanning stock center management, reproductive biology, mathematics, and computer science exemplifying the utility of interdisciplinary research to address practical challenges in experimental biology. Beyond *X. laevis*, the synthetic training strategy and YOLOv10b-based detection models could be adapted to other egg-laying species, positioning this tool as a generalizable solution for image-based classification tasks in developmental and reproductive biology, especially for aquatic species.

## Materials and methods

The *YOLOv10b* architecture by ultralytics [10] was used for model training and testing. The *YOLOv10b* is a variant in the YOLOv10 family of object detectors, designed for improved efficiency and accuracy in real-time object detection tasks. It builds on the core principles of *YOLOv10*, which introduces a unified backbone, neck, and detection head optimized for end-to-end deployment. *YOLOv10b* specifically balances speed and precision by employing a streamlined network structure with fewer parameters and Floating-Point Operations per second (FLOPs) measuring how fast hardware can perform operations compared to larger variants like *YOLOv10l***.** It utilizes advanced architectural components such as decoupled heads for classification and localization, re-parameterized convolutional blocks for efficient inference, and improved label assignment strategies to enhance training stability. The design of *YOLOv10b* makes it particularly suitable for applications where low latency and high accuracy are critical, such as mobile vision systems and edge deployment, a technique in image processing that identifies points with sharp changes in brightness, marking object boundaries or contours. *YOLOv10b* was also selected for a mechanism-based reason directly relevant to our task: its Non-Maximum Suppression (NMS)-free and consistent dual-assignment training removes a documented failure mode of earlier YOLO versions (v5–v9), in which touching or overlapping objects common in embryo clutches are suppressed as duplicate detections, causing systematic under-counting [10].

### Data collection

As part of a multi-year collaborative project between the Aquatic Germplasm and Genetic Resources Center (AGGRC) at the Louisiana State University Agricultural Center and the National *Xenopus* Resource (NXR), a pathway for high-throughput cryopreservation of *X. laevis* sperm was developed [6]. This study was carried out in strict accordance with federal and state laws, regulations, and policies described in the Public Health Service Policy on the Humane Care and Use of Laboratory Animals, the Guide for the Care and Use of Laboratory Animals, and USDA Animal Welfare Regulations. The protocol was approved by the Institutional Animal Care and Use Committee at the Marine Biological Laboratory (Protocol Number: 25−03).

Through continued collaboration with the AGGRC, data consisting of 233 images of early development *X. laevis* embryos were obtained at the NXR following in vitro fertilizations. Of 233 images, 43 were selected for synthetic training, validation and 10 images for the generation of unseen synthetic testing sets, along with additional 20 experimental images from NXR were collected for the independent experimental testing set. Various images of different developmental stages, quality, and conditions were available (Fig 1) of which we chose 53 to create the synthetic dataset based on following criterion including uniform background, in-focus embryos, absence of debris or bubbles, and clear visual separation between fertilized and unfertilized embryos suitable for unambiguous ground-truth labeling.

These images were collected with Nikon SMZ1000 and SMZ1270 stereo microscopes (manufactured by Nikon, Shinagawa, Tokyo, Japan) by holding or mounting a cellphone camera (Google Pixel 5, Santa Clara County, California, United States) to the eyepiece. The images were illuminated with a Fiber-Lite MI-150 High Intensity Illuminator. The stereo microscope objectives were Nikon Plan Apo 1x WF WD:70 and the prior WD:70 model (manufactured in Shinagawa, Tokyo, Japan).

**Fig 1:**
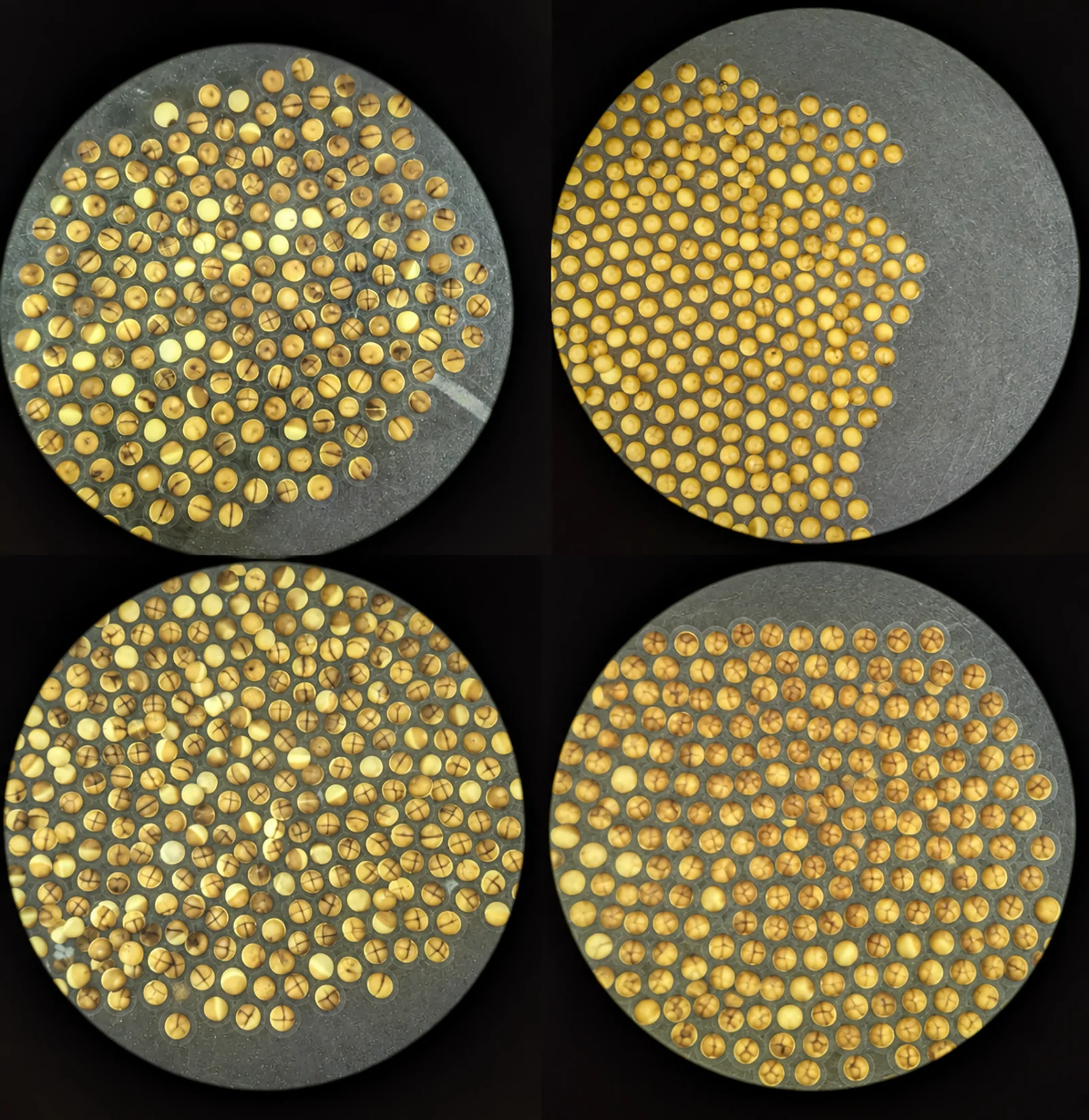
Examples of *X. laevis* eggs and embryos within petri dishes. Images provided by the National *Xenopus* Resource (NXR).

### Generating synthetic data

Synthetic images that closely resembled the original images were created to facilitate model training. The primary reason for synthetic images was to eliminate the need for time-consuming annotation by manually counting and labeling embryos. This approach also allowed us to generate as many images as necessary and control the number of embryos in each image. Ultimately, we aimed to train the neural network on these synthetic images and select the models to be used for training and testing on the original images. To generate the synthetic dataset, original images of fertilized and unfertilized eggs were cropped. The background in the original images, which represented empty space in the petri dishes, were also cropped to enhance the training. The cropped images were obtained using the markup tool of the preview app on a *MacBook Pro M1/M2 Apple Silicon*. Cropped images were saved in a PNG format. We created three different folders: fertilized, unfertilized, and background (Fig 2), to save the respective types of cropped images. The categorization of cropped images was guided by technicians at NXR.

**Fig 2:**
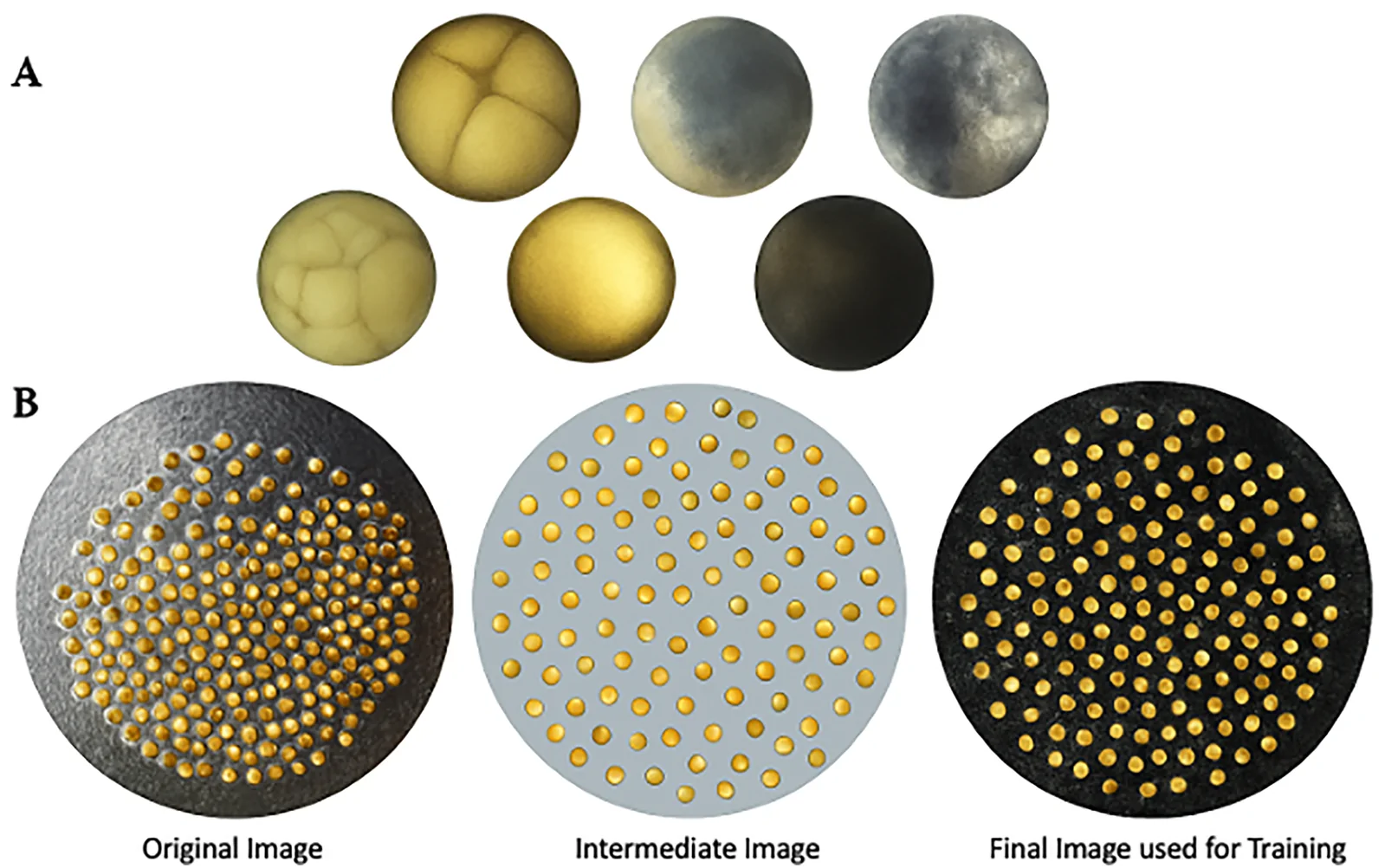
Examples of cropped images of fertilized and unfertilized *X. laevis* eggs and backgrounds, and examples of the process used to generate synthetic images. (A) The embryos with cleavage represents fertilized embryos; middle two with in both the rows represent unfertilized eggs, and third column represents background images. (B) The image in column represents original image acquired from NXR; the Petri dish in the middle column represents the intermediate version of synthetic image which was not used for any training and the third column at the end represents the final synthetic image for used in training and validation. The original images were zoomed in 300% before cropping.

The candidates for the generation of the synthetic embryos were selected using the random python module from the cropped egg images within the fertilized and unfertilized folders. Once an egg was placed in the generated image, another egg was placed adjacent to it at an angle of $\frac{i\pi }{\text{4}}$*,* where *i* an integer and *i* ϵ[1,8]. After the image was populated with about 100–300 eggs and embryos with the fertilization percentage varying between 20–80% with a step size of 20% per image, a background was added by selecting randomly from the background folder. The final image was produced along with the *YOLO* annotations [10,12], which provided the location and size of the object and classification as fertilized or unfertilized. Fertilized eggs were represented as class “0” and unfertilized eggs as class “1.” Parameters of image generation could be controlled, including the percentage of fertilized and unfertilized eggs, the maximum and minimum number of total eggs, egg sizes, and the space between each egg. The code for generating synthetic images is available as a CLI tool on GitHub: https://github.com/Schapagain/scatter-yolo.

### Selecting effective optimizers

Selecting an effective optimizer is crucial for enhancing the performance and efficiency of a machine-learning model. We evaluated the *YOLOv10b* model on four different optimizers using box loss, classification cross entropy loss, and distribution focal loss [10]: Stochastic Gradient Descent (SGD) [13], Adam [14], AdamW [15], and NAdam [16]. Each optimizer was assessed over 100 training epochs, Intersection over Union (IOU) [17] of 0.45, random state 42, and batch size of 16, with an early stopping criterion set to terminate training if there was no improvement in losses for 10 consecutive epochs. This approach ensures that the model did not overfit or waste computational resources [18]. The dropout rate was kept at default of 0.2 at this step [19]. By comparing the convergence rates, stability, and mean average precision at IOU 0.5 (mAP50) [20] of each optimizer, we aimed to determine the most effective optimizer for our application.

### Selecting epoch and IOU for effective optimizer and model training

After the selection of effective optimizers, optimum value of IOU and training epochs needed to be identified. For that, SGD, Adam, and AdamW optimizers were selected for further analysis because of their comparable performances in the previous step. The NAdam was not selected for further analysis because it was not able to generate definite values for training and validation losses for all the epochs. To select the best model for testing, three optimizers — SGD, Adam, and AdamW — were evaluated across 50, 100, and 150 epochs. During the experiment, the batch size was fixed at the default value of 16, initial and final learning rates were fixed at 0.01, random state was 42, and dropout rate was kept at 0.2. Additionally, five distinct types of data augmentation were applied to enhance model robustness [21]: *BGR* color channel weight set to 0.5; *mosaic*: a method that creates a collage of four images [22], at its default value of 1, *mixup*: mixing two images into one to create a new image with modified object labels at 0.5, flip image upside down (*flipud*) and flip left to right (*fliplr*) at 0.5. Other augmentation parameters, such as *hsv_h, hsv_s*, and *hsv_v* [23], were maintained at their default values of 0.015, 0.7, and 0.4, respectively. The input image size was fixed at the default value of 640. For each of the epochs with the configurations as mentioned above, the IOU was varied within the range of 0.1 to 0.8, covering eight distinct IOU values. This comprehensive testing framework allowed the impact of different optimizers, training epochs, IOUs, dropout rates, and augmentation techniques on model performance to be assessed, ultimately guiding the selection of the optimal model configuration for the application.

For each of the models, the training and validation performance was accessed using mAP50, mean average precision at an IOU range [0.5, 0.95] (mAP50-95), recall, box loss (box_loss), classification cross entropy loss (cls_loss), and distribution focal loss (dfl_loss). The mAP50 is the average precision computed at a single IOU threshold of 0.5. The IOU of 0.5 means that the predicted box was considered true positive if the overlap with the ground truth bounding box was at least 50%. On the other hand, the mAP50-95 was the average of average precisions calculated over 10 iterations with varying IOU of 0.5 to 0.95 with steps of 0.05. The mAP50-95 was considered a stricter measure of location accuracy compared to mAP50.

### Benchmark comparison against RT-DETR

To evaluate whether the choice of YOLOv10b was justified over alternative detectors, we performed a direct comparison against *RT-DETR-L* [24] under matched conditions. The large scale real time detection transformer (*RT-DETR-L*) was chosen as a cross-family comparator to test whether the NMS-free advantage that motivated our choice of YOLOv10b generalizes beyond the YOLO family. RT-DETR is a transformer-based detector that is also NMS-free, using set-based Hungarian matching during training rather than the consistent dual-assignment strategy of *YOLOv10b*. Its large variant (~32M parameters) is comparable in capacity to *YOLOv10b* (~19M parameters), and it is supported by the Ultralytics API, allowing the same dataset, splits, augmentation, and evaluation protocol to be applied. Comparing these two models therefore tested whether improvements attributable to NMS-free training were specific to the *YOLOv10b* architecture or reflect a broader architectural advantage across detector families. *RT-DETR-L* was trained on the same 1,194 synthetic images with the same training–validation split used for the *YOLOv10b* experiments described above. Training was performed for 150 epochs using the SGD, Adam, and AdamW optimizers with all other hyperparameters matched to those used for *YOLOv10b* to ensure a fair comparison. All training was performed on high performance computer (HPC) at Louisiana State University using a Tesla V100-PCIE-16GB GPU with a fixed random seed of 42 to reduce run-to-run variance.

### Testing on synthetic and original images

#### Testing with synthetic images.

To generate an additional dataset comprising 120 synthetic images, 50 fertilized embryos, 50 unfertilized eggs, and 10 background samples were extracted from 10 images, not used to generate the training and validation data sets. This approach evaluated the model’s performance on previously unseen data. Within these synthetic images, the number of embryos ranged from 40 to 95, with the proportion of fertilized embryos varying between 20% and 80%. The confusion matrix for the overall synthetic testing set was generated by matching the center coordinates and class labels of the predicted bounding boxes with those of the synthetic test set. The performance of the three models was assessed using metrics such as accuracy, precision, recall, Matthew’s correlation coefficient (MCC), and F1 score.


$$\begin{array}{c} \text{Accuracy}=\frac{\text{TP}+\text{TN}}{\text{FP}+\text{FN}+\text{TP}+\text{TN}} \end{array}$$ (1)

$$\begin{array}{c} \text{Precision}=\frac{\text{TP}}{\text{TP}+\text{FP}} \end{array}$$ (2)

$$\begin{array}{c} \text{Recall}=\frac{\text{TP}}{\text{TP}+\text{FN}} \end{array}$$ (3)

$$\begin{array}{c} \text{MCC}=\frac{\text{TP}\times \text{TN}-\text{FP}\times \text{FN}}{\sqrt{\left(\text{TP}+\text{FP}\right)\left(\text{TP}+\text{FN}\right)\left(\text{TN}+\text{FP}\right)\left(\text{TN}+\text{FN}\right)}} \end{array}$$ (4)

$$\begin{array}{c} \text{F1}=\text{2}\times \frac{\text{Precision}*\text{Recall}}{\text{Precision}+\text{Recall}} \end{array}$$ (5)

Where TP = True Positives; TN = True Negatives; FP = False Positives; FN = False Negatives.

#### Testing with original images.

A separate set of 20 experimental images was supplied by the NXR. To assess the performance of the three *YOLOv10b* models, the number of fertilized embryos and unfertilized eggs in these original images were manually counted. The experimental test set consisted of 20 actual images from the NXR pool not used in training or synthetic-image generation, selected on the basis of embryo density (approximately 300 embryos per dish) to permit reliable manual ground-truth annotation. Embryos located at the edge of the Petri dish, where their full shape could not be captured by the camera, were included in the count if their fertilization status could be visually confirmed. This was done to avoid including embryos that could not be correctly identified by humans and the trained models. The accuracy of prediction was calculated as follows:


$$\begin{array}{c} \text{ECA}=\frac{\left(\text{number of predicted embryos}-\text{misclassified embryos}\right)}{\text{Total number of embryos in the image}}\times \text{ 100}\% \end{array}$$ (6)

Where ECA is Embryo Classification Accuracy

Along with the ECA, Sampling Prediction Accuracy (SPA) was also calculated to show the model’s accuracy in terms of absolute error of predicted fertilization.


$$\begin{array}{c} \text{SPA}=\text{1}-\left|\frac{\text{PFE}-\text{AFE}}{\text{AFE}}\right| \end{array}$$ (7)

Where, PFE is number of predicted fertilized embryos; AFE is actual number of fertilized embryos. The error term was defined by the expression inside of the absolute operator. The SPA represented the closeness of the predicted fertilization in the image to the actual fertilization in that image.

The AFE was calculated using following formula:


$$\begin{array}{c} \text{AFE}=\left(\text{PFE}+\text{FmcU}+\text{FnD}\right)-\text{UmcF} \end{array}$$ (8)

Where, FmcU was number of fertilized embryos misclassified as unfertilized; FnD was number of fertilized embryos not detected by the machine and UmcF is number of unfertilized embryos wrongly classified as fertilized.

### Developing the user interface

A desktop application program was developed to allow users to upload their embryo images and to run prediction models. Specifically, NumPy (v1.25.1) [25] and ultralytics (v8*)* packages were used to support prediction computations, and PyQt6 (≥v6.6.1), along with qtawesome (v1.4.0*)* for icons, were used to build the graphical interface.

The application used a hidden directory in its application path to store images uploaded by the user and stored files generated during the prediction and annotation processes in the same directory. All artifacts stored in this directory were erased when the user uploaded another batch of images to the application, however the user was given an option to save the prediction results and the annotated images to a safe location after every model run.

As prediction and annotation tasks were computationally intensive, running either of them alongside the user interface in a single thread would compromise the responsiveness of the application. This was resolved by using the *QThreadPool* class provided by *PyQt6* to run any tasks in the worker threads. These worker threads communicated with the main application via signals and slots – a messaging mechanism native to *PyQt6*. The application was packaged using *PyInstaller* for MacOS M1/M2 Apple Silicon platforms.

## Results

### Data collection

Some original images had inconsistent lighting, which introduced noise in the cropped images. The images with inconsistent light were selected from the 233 experimental images provided by NXR, to improve the model’s efficacy in classifying objects with varied experimental conditions. In total, images of 200 fertilized embryos, 200 unfertilized eggs, and 100 backgrounds were cropped for the construction of the training set. Various shapes and color variations of embryos were used to enhance the potential of predicting images with divergent conditions. Another set of images with 50 fertilized, 50 unfertilized, and 10 backgrounds were cropped from the 10 images not used in training and validation sets before for the construction of an independent synthetic validation set of 120 images (Fig 2). The construction of the validation set from 10 separate set of images was done to avoid data leakage and to make a clear distinction between the datasets.

### Generating synthetic data

A total of 1,194 synthetic images containing various egg numbers were generated for training purposes (Fig 3). The maximum number of embryos/eggs in the training images was 250, and the minimum number was 25. The percentage of fertilized embryos in the generated images varied between 20% and 80% with step size of 20. The generation was achieved by use of multi-threaded codes, which performed the generation in 4 threads, significantly reducing the time of image generation. It took approximately 30 minutes to generate 1,194 images in Apple MacBook Pro with Silicon M1 chip with 8GB RAM. Of the 1,194 images, 1,134 images with 100 background images were used in the training set, and remaining 60 synthetic images were used for the validation set.

**Fig 3:**
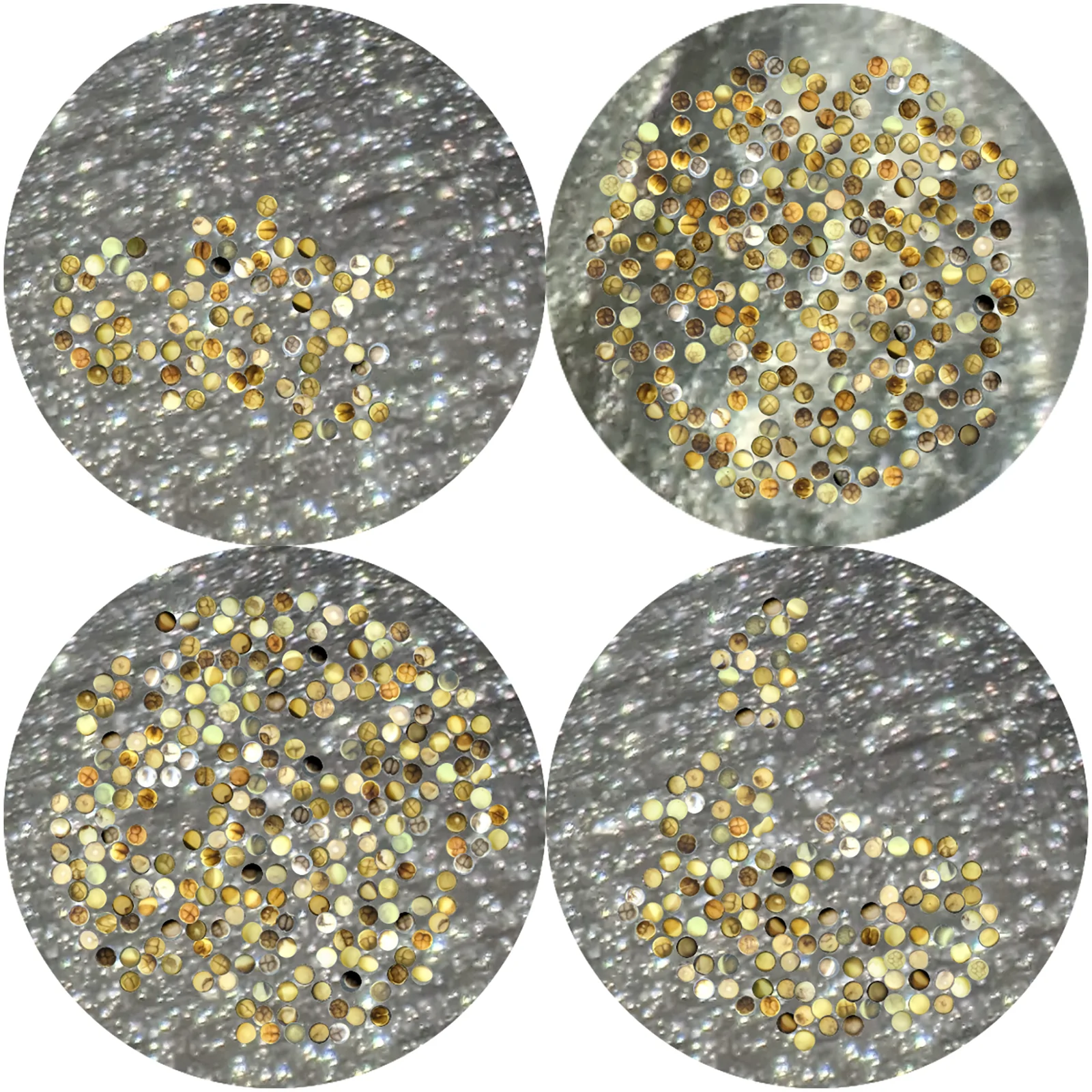
Examples of the generated synthetic images with different ratios of fertilized embryos to unfertilized *X. laevis* eggs. These synthetic images were produced using the scatter-yolo command line tool (CLI).

### Selecting effective optimizers

Four optimizers were tested with the parameters described in Materials and Methods. For each of these optimizers we collected the loss metrics (Table 1). Based on lower losses and high precision and recall, three optimizers (i.e., Adam, SGD, and AdamW) were selected from the initial set of four. Adam and AdamW exhibited comparable training and validation box losses, with a mAP50 of 0.995. The SGD optimizer was selected for its notably lower training and validation box losses, while maintaining the same mAP50 value of 0.995. The Adam, AdamW and SGD optimizers were selected based on the mAP50 values for these optimizers.

**Table 1:** Optimizer selection based on performance measured in terms of losses, precision, recall and mAP50 for four different optimizers. The Adam, AdamW and SGD optimizers were selected based on the highest values of performance metrics and lowest losses. Model trainings were setup for 100 epochs with early stopping criterion if there was no improvement in the performance was seen for ten consecutive epochs.

Optimizer	Epoch	Training box loss	Validation box loss	Training class loss	Validation class loss	Training focal loss	Validation focal loss	Precision	Recall	mAP50
Adam	27	0.699	0.474	0.455	0.275	1.56	1.53	0.997	0.998	0.995
AdamW	29	0.608	0.425	0.385	0.247	1.55	1.52	0.997	0.998	0.995
NAdam	26	1.21	NA	0.825	NA	1.61	NA	0.456	0.046	0.257
SGD	82	0.505	0.259	0.298	0.168	1.55	1.52	0.998	0.998	0.995

We also created a visualization of the box loss, class loss, and distribution focal loss (DFL) across training epochs for the Adam, AdamW and SGD optimizers. Box loss quantifies how well predicted bounding boxes align with ground truth, where lower values indicate more accurate object localization (Fig 4A, B, C). Class loss evaluates the correctness of object label assignments (Fig 4D, E, F) while focal loss enhances bounding box precision by treating coordinate regression as a classification problem over discretized distances, mitigating class imbalance using principles from the focal loss function (Fig 4G, H, I).

**Fig 4:**
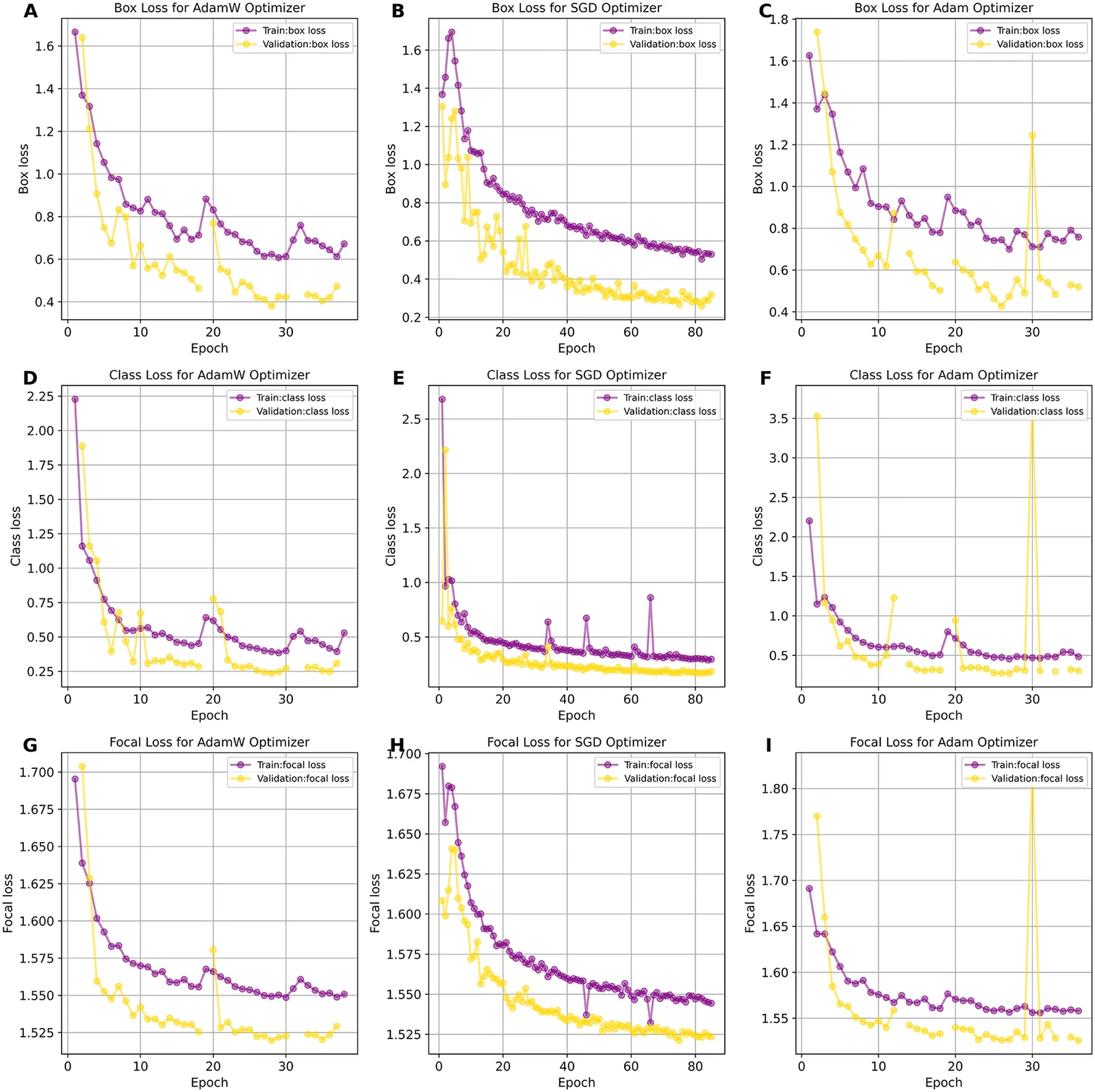
Representation of losses for the optimizers. Top row represents the box loss for (A) AdamW, (B) SGD, and (C) Adam. Middle row represents the class loss for (D) AdamW, (E) SGD, and (F) Adam. The bottom row represents the focal loss for (G) AdamW, (H) SGD, and (I) Adam optimizers. The x-axis of each subplot represents Epoch before training was terminated due to early stopping and y-axis represents the loss metric. In the figure training losses are in purple and validation losses are represented in orange.

All three optimizers yielded models with consistently high precision and recall of 0.997, and 0.998 underscoring their ability to accurately detect and correctly classify embryos/eggs in the images (Fig 5). High precision reflects a low false-positive rate, whereas high recall indicates effective detection of true positives. This favorable balance is a hallmark of the YOLO architecture’s unified detection framework, which employs a single forward pass, optimized anchor boxes, enhanced feature extraction, and decoupled prediction heads. In the specific context of classifying fertilized versus unfertilized *X. laevis* eggs, these high-performance metrics enabled robust and reliable automated analysis, reducing the need for manual verification. The performance metrices for NAdam model was the lowest among all the optimizers with precision 0.456 and recall was 0.046 (S1 Fig). Because the NAdam model demonstrated low performance metrics, it was excluded from further analyses.

**Fig 5:**
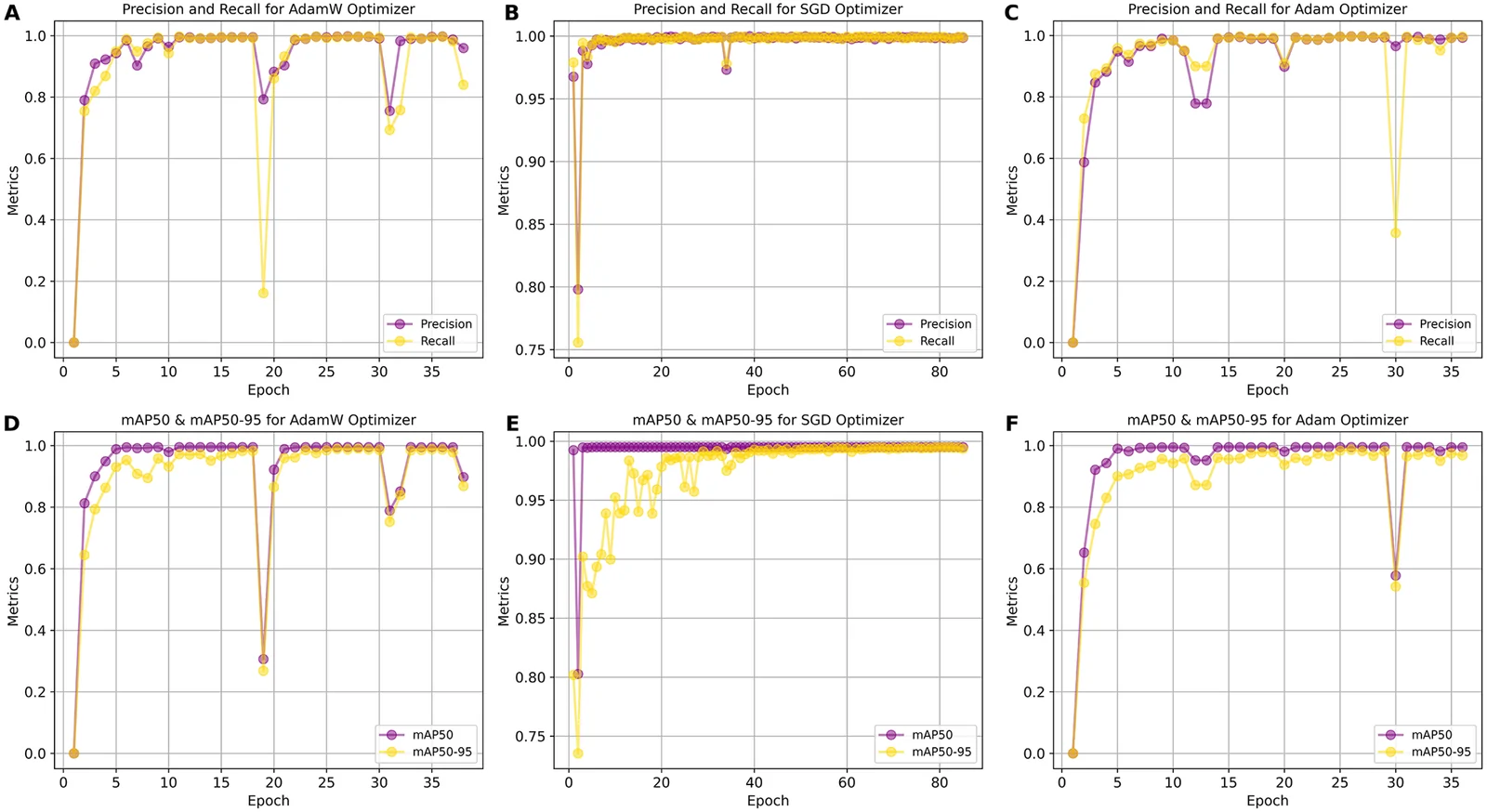
Training and validation performance metrices precision, recall, mean average precision at 50% IOU (mAP50) and ranged mAP50-95. Top row represents the precision (purple) and recall (orange) for (A) AdamW, (B) SGD and (C) Adam optimizer models. Bottom row represents mAP50 (purple and mAP50-95 (orange) for (D) AdamW, (E) SGD and (F) Adam optimizer models.

### Selecting epoch and IOU values for effective optimizer and model training

After selecting the optimizers, we aimed to identify the optimal combination of epoch and IOU values for each optimizer model. To achieve this, *YOLOv10b* models were trained for 50, 100, and 150 epochs using eight different IOU thresholds ranging from 0.1 to 0.8. Lower IOU values can result in overly lenient bounding boxes, potentially compromising prediction quality, while higher IOU values (e.g., > 0.7) may enforce overly strict criteria, leading to increased false negatives. Therefore, our goal was to determine the most balanced IOU threshold that yields high prediction accuracy.

Based on training and cross-validation outcomes, the best-performing configurations with the lowest box losses and highest mAP50 recall were achieved at epoch 150 using IOUs of 0.3 for the AdamW optimizer, 0.5 for Adam, and 0.5 for SGD. Inspection of the training and validation loss curves for these three configurations (Fig 6, panels A–I) further supported this selection. Across all three optimizers, box, class, and focal (DFL) losses declined steadily over the 150 epochs and plateaued toward the end of training, with validation losses tracking training losses closely and remaining consistently at or below training loss throughout indicating no evidence of overfitting. The SGD run exhibited the smoothest convergence profile compared to Adam and AdamW. By epoch 150, final training box losses had converged to 0.297 (AdamW), 0.322 (Adam), and 0.260 (SGD), with corresponding validation box losses of 0.227, 0.252, and 0.230, respectively.

**Fig 6:**
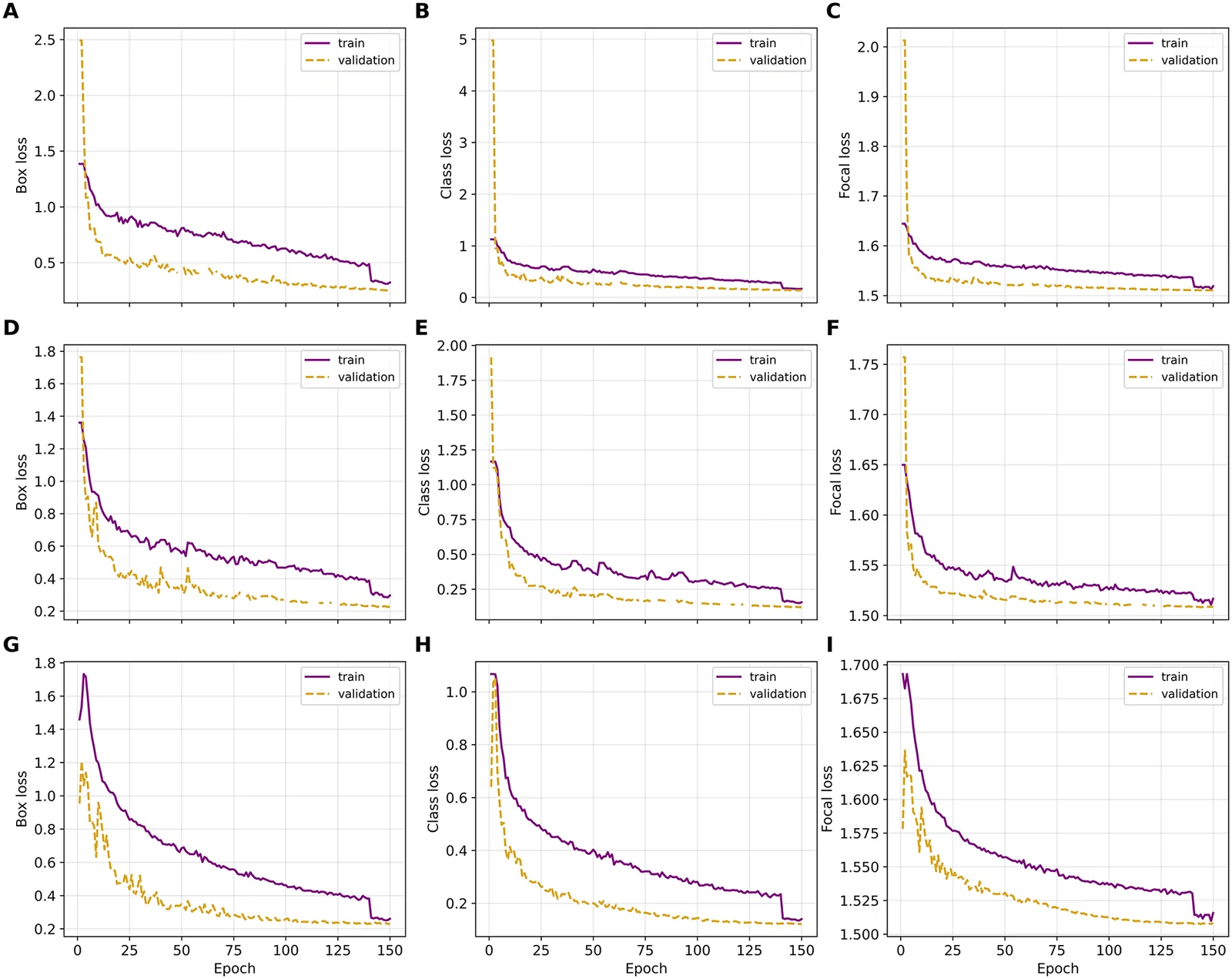
Training and validation loss curves for the three selected *YOLOv10b* configurations. Box loss (left column), class loss (middle column), and focal (DFL) loss (right column) are shown across 150 training epochs for each optimizer: (A–C) Adam with IOU = 0.5, (D–F) AdamW with IOU = 0.3, and (G–I) SGD with IOU = 0.5. Training loss is shown in purple (solid) and validation loss in gold (dashed). All three optimizers exhibit monotonically decreasing losses, with validation loss tracking training loss closely and remaining at or below training loss throughout, indicating no evidence of overfitting. The SGD configuration displays the smoothest convergence profile across all three loss components.

Due to the close similarity in precision, recall, and mAP50 values across different settings for each optimizer, final model selection was based on the lowest observed training loss values of 0.297 (AdamW), 0.322 (Adam), and 0.260 (SGD) (S1 Table). Because all three models trained for 150 epochs demonstrated comparable performance, they were each selected for validation on independent synthetic and original datasets. A detailed list of metrics containing all combinations of epoch and IOU values explored is available at https://osf.io/fm9st.

### Benchmark comparison against RT-DETR

To assess whether the detection performance of *YOLOv10b* was specific to its architecture or reflected properties shared with other NMS-free detectors, we compared it against RT-DETR-L trained under same conditions as *YOLOv10b*. Because *RT-DETR* uses set-based Hungarian matching rather than the box/class/DFL loss decomposition of YOLO, box loss and focal loss values are not directly comparable across the two architectures and are reported only for *YOLOv10b*; classification loss, precision, recall, and mAP@0.5 are computed identically for both detectors and are reported for both.

Both detectors achieved essentially equivalent performance on the synthetic validation set. Precision, recall, and mAP@0.5 differed by no more than 0.008 between the two architectures across the tested optimizers (Table 2). *YOLOv10b* reached precision, recall, and mAP@0.5 of 0.999, 0.999, and 0.995 respectively under all three optimizers (Adam, AdamW, SGD). *RT-DETR-L* reached 0.997 / 0.998 / 0.995 under Adam and 0.991 / 0.989 / 0.994 under SGD. AdamW failed to reach accuracy at 150 epoch. Classification loss was somewhat higher for RT-DETR-L than for *YOLOv10b* (training class loss 0.211 vs. 0.140–0.168; validation class loss 0.168 vs. 0.120–0.138). This supports our original mechanistic justification for choosing an NMS-free detector, while indicating that *YOLOv10b* and *RT-DETR* exhibit similar performance. But, we retained *YOLOv10b* as the deployed detector because it has substantially fewer parameters (~19M versus ~32M for *RT-DETR-L*), reducing GPU memory requirements and inference latency for end users running the tool on standard lab hardware without compromising accuracy**.**

**Table 2:** Comparison of training and validation performance between *YOLOv10b* and *RT-DETR-L* across three optimizers.

Method	Optimizer	IOU	Train Box Loss	Validation Box Loss	Train Class Loss	Validation Class Loss	Train Focal Loss	Validation Focal Loss	Precision	Recall	mAP50
YOLOv10b	Adam	0.5	0.322	0.252	0.168	0.138	1.519	1.510	0.999	0.999	0.995
	AdamW	0.3	0.297	0.227	0.156	0.120	1.517	1.509	0.999	0.999	0.995
	SGD	0.5	0.260	0.230	0.140	0.121	1.519	1.510	0.999	0.999	0.995
RT-DETR-L	Adam	–	–	–	0.211	0.168	–	–	0.997	0.998	0.995
	AdamW	–	–	–	–	–	–	–	–	–	–
	SGD	–	–	–	0.226	0.190	–	–	0.991	0.989	0.994

### Validation of unseen data

We used an independent validation strategy which tested the performance of all the models on 120 synthetic images generated as before and 20 original experimental images from NXR. These experimental images were not used during the generation of the synthetic images.

#### Testing with synthetic images.

We validated the performance of each model using the best weights saved during training. On the synthetic image set consisting of 120 images, the mean percentage accuracies of models (calculated using eq. 1) employing was 87% for the Adam optimizer, 89% for AdamW, and 90% for SGD. The F1 scores for these models ranged from 0.85 to 0.89. The F1 score represents the harmonic mean of the precision and recall, and it represents the balance between these quantities. An F1 value of between 0.7 to 0.9 is generally considered as an acceptable measure for the balance [26,27]. The models with F1 values in this range can be considered reliable for most applications in machine learning [28]. Independent validation of the three models revealed that they could learn egg and embryo features and determine fertilization status accurately. The high precision of greater than 0.98, recall values greater than 0.74 and F1 value 0.854 represent that all the models could accurately identify fertilized embryos vs unfertilized eggs (Table 3). Precision, recall, MCC and F1 values were calculated with the help of equation 2–5 in methods section.

**Table 3:** Percent accuracy, Precision, Recall, MCC and F1 values of Adam, AdamW and SGD models calculated over synthetic independent validation set of 120 images (MCC: Matthew’s Correlation Coefficient).

Model	Accuracy (%)	Precision	Recall	MCC	F1
Adam	87.29	0.997	0.746	0.769	0.854
AdamW	89.36	0.986	0.797	0.801	0.881
SGD	89.97	0.994	0.803	0.814	0.888

Among all three models, SGD gave the highest mean accuracy and recall along with comparable precision to the Adam optimizer. The SGD model also had the highest Matthew’s correlation coefficient which is an indirect indicator of superior model performance.

#### Independent validation with original images.

To evaluate the robustness of all three models on actual experimental data, we curated a set of 20 independent laboratory-captured images that were not used during synthetic dataset generation. These images each contained between 169 and 326 embryos, with a median count of 262. On this test set, the Adam model (IOU 0.3) achieved a mean embryo classification accuracy (ECA) of 96 ± 3% and a median ECA of 97%. Its mean (98 ± 2%) and median sampling prediction accuracy (SPA; 99%) were high. The SGD model yielded mean and median ECA values of 96 ± 3% and 96%, and SPA values of 97% ± 2 and 97%. The AdamW model showed a slightly lower performance, with a mean and median ECA of 94 ± 6% and 95%. Its SPA had a mean of 89 ± 22% and a median of 97%. Full per-image results are provided in S2 Table, and summary statistics are reported in Table 4.

**Table 4:** Mean performance of Adam, AdamW and SGD models based on mean percentage embryo classification accuracy (ECA), mean percentage sample prediction accuracy (SPA) based on the 20 experimental images.

Models	ECA (%)	SPA (%)
Adam	96.36 ± 2.87	98.25 ± 2.10
AdamW	94.43 ± 5.56	88.51 ± 21.90
SGD	96.13 ± 2.51	96.79 ± 2.25

ECA and SPA calculations were done using equation 6 and 7, respectively.

### Developing user interface

We developed a GUI-based application called “Froggle (https://osf.io/fm9st),” that can be installed on a computer by downloading and installing the software package. This app allows users to upload a ZIP file containing images of *X. laevis* embryos. After the file was uploaded, the user could select a model to classify the embryos as fertilized or unfertilized in the uploaded images. Different models were available with different optimizers: Adam, AdamW, and SGD. The default mode was set to the optimizer SGD, as it has shown the best performance in terms of accuracy. Once the model had finished running, a table displaying counts of fertilized and unfertilized eggs was shown (Fig 7). Users also had the option to export the table as a CSV file to facilitate data management. After the table was displayed, the model began showing annotated images with bounding box for manual verification.

**Fig 7:**
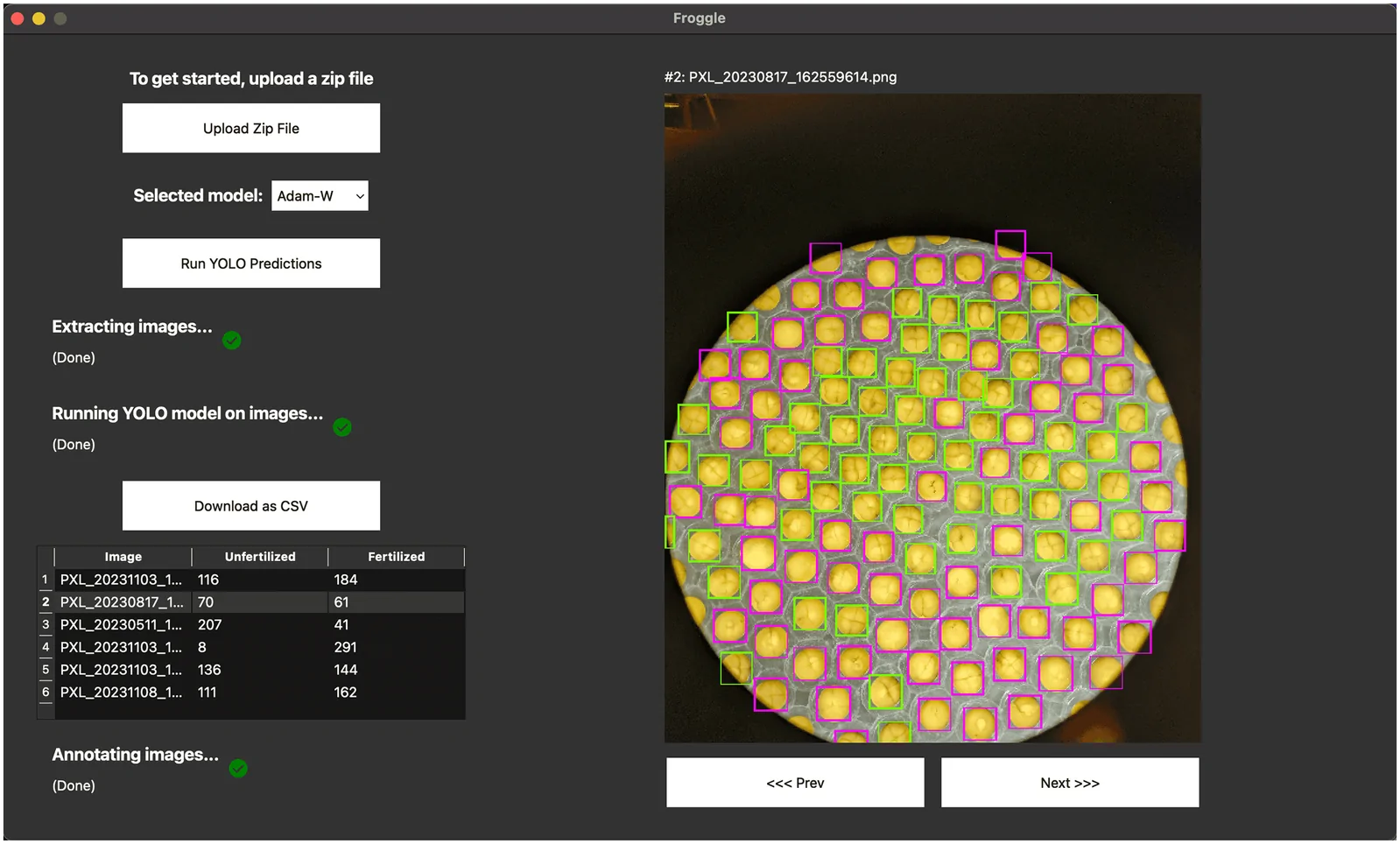
Example output from the Froggle GUI following prediction using AdamW optimizer. A ZIP file containing images of Petri dishes with *Xenopus laevis* embryos can be uploaded through the interface. Prior to use, one of three available prediction models can be selected to analyze the uploaded images. The right panel displays the resulting predictions, including bounding boxes around each detected embryo or egg.

## Discussion

The motivation of the work is a long-term vision to develop technological and innovative infrastructures to support national resource centers and biomedical research communities to better safeguard genetic resources through efficient and reproducible germplasm repositories. These centers typically have static budgets and must continually find new efficiencies of operation to offset cost increases that accrue over time. In this study, an advanced convolutional neural network (CNN) technique was used to automate the detection of fertilized and unfertilized *X. laevis* eggs in easily captured images. The tools developed employed the *YOLOv10b* architecture, a CNN-based method that demonstrated fully acceptable accuracy in distinguishing fertilized from unfertilized eggs, a task traditionally performed manually. This tool was placed within an intuitive graphical user interface to assist availability and use. Widespread adoption of tools such as these offers increased efficiency, which is extremely important in centers with static budgets, and supports community-level standardization in data collection and reporting.

We developed a training approach by creating synthetic images to eliminate the need for manual annotation of the embryos. Synthetic images were generated using cropped images of fertilized embryos and unfertilized eggs and Petri dish backgrounds. It is well-established that machine learning and artificial intelligence (AI) techniques benefit from large, annotated data sets. Our approach accelerated data generation, facilitating access to large amounts of data in a shorter time, which is essential for machine learning training [29–31]. Experiments were conducted to determine the optimal parameters and hyperparameters for CNN training, highlighting the importance of effective optimizer selection for the performance of *YOLOv10b* models.

Optimizing the IOU metric was necessary to maximize correct object detections. Models were trained with IOUs ranging from 0.1 to 0.8 with steps of 0.1, and epochs varying from 50 to 150 with steps of 50, along with various other parameters. This rigorous process identified the best models for each optimizer (Adam, AdamW, and SGD). Optimizing IOU is crucial for detecting objects at specific locations. The models exhibited different accuracy and detection rates due to varying optimal IOU values, with Adam using a threshold of 0.5, AdamW using 0.3, and SGD using 0.5. According to ultralytics, a higher IOU cut-off leads to more detections, potentially increasing false positives. Therefore, it is recommended that users test these models on sample images based on specific requirements before selecting parameters and models for future use.

The comparison with RT-DETR-L showed that both NMS-free detectors achieved essentially equivalent detection performance on this task, supporting our interpretation that the NMS-free training strategy is the architectural feature responsible for reliable detection of touching or overlapping embryos, rather than any specific property of the *YOLOv10b* architecture. *YOLOv10b* was retained as the deployed detector for its substantially lower parameter count (~19M versus ~32M), which reduces memory and latency requirements for end users.

Across all three optimizers, precision was consistently near-perfect (0.986–0.997) while recall was lower (0.746–0.803), indicating that the models rarely report false detections but miss a fraction of true embryos. Because this pattern is stable across Adam, AdamW, and SGD, and because both training and validation sets are drawn from the same synthetic generator, we attribute the recall shortfall to factors intrinsic to the pipeline and evaluation rather than to optimizer choice. First, our compositing procedure occasionally produced embryos that partially occluded one another; the underlying embryo remains in the ground-truth annotation but is visually inaccessible, contributing an irreducible floor of false negatives that reduced recall without affecting precision. Second, YOLOv10’s NMS-free one-to-one detection head produced sharp confidence distributions, so a subset of true embryos received scores just below the detection threshold and were counted as misses even though the model has partially learned them. Third, at the mAP@0.5 evaluation criterion, predicted boxes with less than 50% IOU with ground truth were counted as false negatives even when the embryo had been correctly localized in approximate position which could be a source of recall depression for small, densely packed embryos. Class imbalance between fertilized and unfertilized embryos in the training set may have contributed additional per-class differences. Importantly, the counting-accuracy metric was the relevant quantity for a laboratory tool that reached 94–96% accuracy across optimizers, indicating that box-level misses do not translate into large errors in total embryo counts on original images from labs, likely because missed detections were approximately balanced across the two classes.

A graphical user interface (GUI) application named “Froggle” was developed to facilitate use of the tool developed herein. Implementing a multithreaded approach ensured the application would remain responsive during computationally intensive tasks, enhancing user experience. This application supports batch image uploads, model selection, and visualization of fertilization ratios, allowing researchers to quantify embryo viability with minimal effort. The entire workflow — from data generation to model deployment — has been released as open-source software, fostering reproducibility and improvement through a community-based approach.

This method of identifying the fertilization status of frog eggs using a synthetically generated training dataset represents the first of its kind in this field. The present study presents a novel method, and it was intended to provide a solid foundation for further advancement. For example, the next step would be to expand the model scope to differentiate across the full range of developmental stages for embryos (e.g., NF1 – NF18) [32] at various temperatures.

Previously, the temporal development of *X. laevis* embryos and presence of various surface lipids was investigated using time-of-flight secondary ion mass spectrometry (ToF-SIMS) [33]. Accurate identification of embryo developmental stages would significantly facilitate execution and interpretation of these types of experiments. The ability to plan experiments precisely and with ease would aid in preserving samples through snap freezing, allowing the preservation of specific developmental stages for detailed study. Because embryo development in ectotherms is temperature dependent, it is possible to speed or slow processes to reach a desired stage of development at a precise time [34,35]. Combining published knowledge of temperature and growth with a program that identifies and predicts developmental stages could streamline workflow for researchers. Delineation of these workflows by use of tools such as process mapping and simulation modeling can further enhance efficiency and reduce costs and bottlenecks [36]. Our methodological approach is highly versatile and could be adapted to other aquatic animal models such as zebrafish, axolotls, *Aplysia,* and aquaculture species such as catfish or oysters. This work was developed as open source, with all codes made publicly accessible to foster collaboration across the broader research community.

Going forward, it will be increasingly important to work in multidisciplinary and interdisciplinary teams to ensure that design and prototyping will produce practical outcomes with relevance to users in other fields, which requires incorporation of user experience evaluation [37]. This project was initiated under the framework of the Math Capstone Project at the Louisiana State University Department of Mathematics [38], which was designed to promote collaboration among students and professionals from various academic disciplines. With the support of an interdisciplinary environment at AGGRC this team comprised graduate students, industry volunteers, and faculty members from diverse fields including biomedicine, computational biology, mathematics, and computer science each bringing unique skills essential for success. The computational biology students contributed their expertise in developing machine learning models, while the mathematics students focused on optimizing the algorithms and statistical methods critical to refining model accuracy. The computer science students were instrumental in coding scatter yolo to generate synthetic image datasets and creating the user-friendly graphical interface that made the tool accessible to biomedical researchers without computational backgrounds.

Biologists from the NXR and AGGRC provided essential knowledge about amphibian biology and development of germplasm repositories. Their insights ensured the project remained application relevant and helped validate the performance on actual experimental data. Collaboration with the NXR provided high-quality biological images and expert consultation on the operation of resource centers and research laboratories. This interdisciplinary collaboration allowed the project to tackle complex biological questions with innovative solutions that would have been challenging or impossible by sole reliance on expertise from individual fields, underscoring the importance of interdisciplinary collaboration in technological innovation, especially with aquatic species [39].

This study represents a significant advancement in the potential automation of embryo stage identification in *X. laevis*. By developing a robust and accurate YOLO-based CNN tool and validating its performance on synthetic and actual datasets. We demonstrated the potential to streamline and enhance experimental research involving *X. laevis*. The availability of the data and code, along with the user-friendly interface, supports practical implementation and refinement. In the future research, we would focus on testing and improving this tool through user interaction, reproducibility assessment [40]. This study provides a foundation for development of comprehensive platforms for quality assessment and quality control for germplasm repository networks for biomedical research models [41]. Along with this, study also lays the foundation to develop artificial intelligence based tools in future for agricultural and conservation use.

## Supporting information

S1 Fig Training and validation losses and performance metrics precision, recall, mAP50 and mAP50–95 for NAdam optimizer model. In the top row, each column from left to right represents box loss, class loss and focal loss, respectively. In the top row, the purple scatter line plots represent loss values calculated during training and gold line-dots represent validation losses. The bottom left Figrepresents precision (purple) and recall (gold) of NAdam model. The bottom right scatter line plot represents the mAP50 (purple) and mAP50–95 (gold) values.
(PNG)

S1 Table Optimizer selection based on performances measured in terms of losses, precision, recall and mAP50 for four different optimizers Adam, AdamW, SGD and NAdam trained for 100 epochs. The Adam, AdamW and SGD optimizers were selected based on the highest values of performance metrics and lowest losses.
(DOCX)

S2 Table Comprehensive results of independent testing on 20 original images with Adam, AdamW and SGD models representing embryo classification accuracy (ECA), error of predicted fertilization (Error) and sampling prediction accuracy (SPA) calculated by subtracting error from 1. The table also captures the mean and median number of embryos, number of actual fertilized embryos, predicted embryos and miss-classifications.
(XLSX)
