# Supplementary material for: Evaluation of machine learning models for classification of fertilization status of African clawed frog (xenopus laevis) by use of synthetic images
Source: PLoS One. 2026 Nov 11;21(11):e0360384. doi: 10.1371/journal.pone.0360384 (PMC13754134; doi:10.1371/journal.pone.0360384)
Supplement: Supplementary file 1 [file pone.0360384.s002.docx]

**S1 Table**. **Optimizer selection based on performances measured in terms of losses, precision, recall and mAP50 for four different optimizers Adam, AdamW, SGD and NAdam trained for 100 epochs**. The Adam, AdamW and SGD optimizers were selected based on the highest values of performance metrics and lowest losses.

| **Optimizers** | **Epoch** | **IOU** | **Train Box Loss** | **Validation Box Loss** | **Train Class Loss** | **Validation Class Loss** | **Train Focal Loss** | **Validation Focal Loss** | **Precision** | **Recall** | **mAP50** |
| --- | --- | --- | --- | --- | --- | --- | --- | --- | --- | --- | --- |
| Adam | 50 | 0.3 | 0.3948 | 0.2846 | 0.2052 | 0.1628 | 1.521 | 1.512 | 0.9991 | 0.9990 | 0.995 |
|  | 100 | 0.1 | 0.3478 | 0.2630 | 0.1827 | 0.1462 | 1.515 | 1.511 | 0.9990 | 0.9998 | 0.995 |
|  | 150 | 0.5 | **0.3225** | 0.2520 | 0.1688 | 0.1379 | 1.519 | 1.510 | 0.9998 | 0.9995 | 0.995 |
| AdamW | 50 | 0.7 | 0.4056 | 0.2677 | 0.2137 | 0.1512 | 1.521 | 1.512 | 0.9990 | 0.9993 | 0.995 |
|  | 100 | 0.7 | 0.3554 | 0.2447 | 0.1828 | 0.1315 | 1.514 | 1.510 | 0.9995 | 0.9996 | 0.995 |
|  | 150 | 0.3 | **0.2968** | 0.2266 | 0.1558 | 0.1200 | 1.517 | 1.509 | 0.9998 | 0.9999 | 0.995 |
| SGD | 50 | 0.2 | 0.3504 | 0.2729 | 0.1879 | 0.1496 | 1.519 | 1.512 | 0.9996 | 0.9999 | 0.995 |
|  | 100 | 0.3 | 0.2902 | 0.2445 | 0.1550 | 0.1319 | 1.512 | 1.509 | 0.9993 | 0.9997 | 0.995 |
|  | 150 | 0.5 | **0.2603** | 0.2305 | 0.1401 | 0.1214 | 1.516 | 1.508 | 0.9996 | 0.9996 | 0.995 |
