# Supplementary material for: Evaluation of machine learning models for classification of fertilization status of African clawed frog (xenopus laevis) by use of synthetic images
Source: PLoS One. 2026 Nov 11;21(11):e0360384. doi: 10.1371/journal.pone.0360384 (PMC13754134; doi:10.1371/journal.pone.0360384)
Supplement: Supplementary file 2 [file pone.0360384.s005.docx]

***When submitting your revision, we need you to address these additional requirements.***

***1.Please ensure that your manuscript meets PLOS ONE's style requirements, including those for file naming. The PLOS ONE style templates can be found at***

[***https://journals.plos.org/plosone/s/file?id=wjVg/PLOSOne_formatting_sample_main_body.pdf***](https://journals.plos.org/plosone/s/file?id=wjVg/PLOSOne_formatting_sample_main_body.pdf) ***and***

[***https://journals.plos.org/plosone/s/file?id=ba62/PLOSOne_formatting_sample_title_authors_affiliations.pdf***](https://nam04.safelinks.protection.outlook.com/?url=https%3A%2F%2Ftrack.editorialmanager.com%2FCL0%2Fhttps%3A%252F%252Fjournals.plos.org%252Fplosone%252Fs%252Ffile%253Fid%3Dba62%252FPLOSOne_formatting_sample_title_authors_affiliations.pdf%2F1%2F010f019f73d9f255-dea6c799-6c0d-4c09-8b66-de605db5cf1a-000000%2FlEjMA95ItjqZXPh6d7k6WRVOo3_PnQ3ZDyHbyTsOKfw%3D258&data=05%7C02%7Cgopal.srivastava%40lsu.edu%7C8a0f4700cc354f0bdd1108dee4f76930%7C2d4dad3f50ae47d983a09ae2b1f466f8%7C0%7C0%7C639199948800500789%7CUnknown%7CTWFpbGZsb3d8eyJFbXB0eU1hcGkiOnRydWUsIlYiOiIwLjAuMDAwMCIsIlAiOiJXaW4zMiIsIkFOIjoiTWFpbCIsIldUIjoyfQ%3D%3D%7C0%7C%7C%7C&sdata=xNRc3HfHty8QA3m7cmjfAxVxFGqoFjDRgiEXlRiKtaI%3D&reserved=0)

**Response:** We thank the editor the comment and pointing us towards the journal guidelines regarding formatting of the manuscript. We have made changes to the manuscript according to the guidelines provided and the manuscript is now in compliance with the formatting guidelines. All comments from editors and reviewers have been addressed.

2. ***Thank you for stating the following financial disclosure:***

***“This work was supported in part by the National Institutes of Health, Office of Research Infrastructure Programs (R24-OD028443, R24-OD037797, and R24-OD034058) and National Science Foundation (Award 2229680), with additional support provided by the National Institute of Food and Agriculture, United States Department of Agriculture (Hatch project LAB94420), Louisiana State University Agricultural Center Pilot Program for Enhancement of External Competitive Funding. The Department of Mathematics and Math Capstone Course supported the salaries of M.P. and K.B., and LSU HPC provided support for computational resources. Work at the NXR was supported by the National Institutes of Health Grant P40OD010997. This manuscript was approved for publication by the Louisiana State University Agricultural Center”***

***Please state what role the funders took in the study.  If the funders had no role, please state: "The funders had no role in study design, data collection and analysis, decision to publish, or preparation of the manuscript."***

**Response:** Thank you for pointing it out. We have removed the funding statement from the acknowledgement and moved it to funding statement. We have also added “The funders had no role in study design, data collection and analysis, decision to publish, or preparation of the manuscript” in the funding statement.

**3. *Thank you for stating the following in the Acknowledgments Section of your manuscript:***

***“This work was supported in part by the National Institutes of Health, Office of Research Infrastructure Programs (R24-OD028443, R24-OD037797, and R24-OD034058) and National Science Foundation (Award 2229680), with additional support provided by the National Institute of Food and Agriculture,  United States Department of Agriculture (Hatch project LAB94420), Louisiana State University Agricultural Center Pilot Program for Enhancement of External Competitive Funding. The Department of Mathematics and Math Capstone Course supported the salaries of M.P. and K.B., and LSU HPC provided  support for computational resources. Work at the NXR was supported by the National Institutes of Health Grant P40OD010997. This manuscript was approved for publication by the Louisiana State University Agricultural Center”***

***We note that you have provided funding information that is not currently declared in your Funding Statement. However, funding information should not appear in the Acknowledgments section or other areas of your manuscript. We will only publish funding information present in the Funding Statement section of the online submission form.***

***Please remove any funding-related text from the manuscript and let us know how you would like to update your Funding Statement. Currently, your Funding Statement reads as follows:***

***“This work was supported in part by the National Institutes of Health, Office of Research Infrastructure Programs (R24-OD028443, R24-OD037797, and R24-OD034058) and National Science Foundation (Award 2229680), with additional support provided by the National Institute of Food and Agriculture, United States Department of Agriculture (Hatch project LAB94420), Louisiana State University Agricultural Center Pilot Program for Enhancement of External Competitive Funding. The Department of Mathematics and Math Capstone Course supported the salaries of M.P. and K.B., and LSU HPC provided support for computational resources. Work at the NXR was supported by the National Institutes of Health Grant P40OD010997. This manuscript was approved for publication by the Louisiana State University Agricultural Center”***

***Please include your amended statements within your cover letter; we will change the online submission form on your behalf.***

**Response**: We thank editor for the comment and following the guidance from editor, we have removed the funding statement from the acknowledgement and also added it in cover letter for review by editor.

**4. *In the online submission form, you indicated that “The data used to generate the synthetic images will be made available on request. All codes used to generate datasets and perform training, and validation can be found at*** [***https://github.com/mpande2/EmbryoDetection***](https://github.com/mpande2/EmbryoDetection) ***(accessed on May 5, 2026). The precompiled version of the Froggle app can be accessed at*** [***https://github.com/Schapagain/froggle***](https://github.com/Schapagain/froggle) ***(accessed on May 5, 2026). The compiled version of the Froggle GUI app can be accessed at*** [***https://osf.io/fm9st***](https://osf.io/fm9st) ***(accessed on May 5, 2026). The code for generating synthetic images is available as a CLI tool on GitHub*** [***https://github.com/Schapagain/scatter-yolo***](https://github.com/Schapagain/scatter-yolo) ***(accessed on May 5, 2026).”***

***All PLOS journals now require all data underlying the findings described in their manuscript to be freely available to other researchers, either 1. In a public repository, 2. Within the manuscript itself, or 3. Uploaded as supplementary information.***

***This policy applies to all data except where public deposition would breach compliance with the protocol approved by your research ethics board. If your data cannot be made publicly available for ethical or legal reasons (e.g., public availability would compromise patient privacy), please explain your reasons on resubmission and your exemption request will be escalated for approval.***

**Response**: We have added all the images used to generate the synthetic image dataset has been made publicly available in <https://osf.io/fm9st>. We have also provided all the synthetic training and validation set along with the 20 experimental images used in the unseen dataset.

***5. Please upload a new copy of Figure 4 and 5 as the detail is not clear. Please follow the link for more information:***  [***https://journals.plos.org/plosone/s/figures***](https://nam04.safelinks.protection.outlook.com/?url=https%3A%2F%2Ftrack.editorialmanager.com%2FCL0%2Fhttps%3A%252F%252Fjournals.plos.org%252Fplosone%252Fs%252Ffigures%2F1%2F010f019f73d9f255-dea6c799-6c0d-4c09-8b66-de605db5cf1a-000000%2FERS41R7SyCXv6rmxSPC0rw20oWxM5xnMCPRFpsjx6BE%3D258&data=05%7C02%7Cgopal.srivastava%40lsu.edu%7C8a0f4700cc354f0bdd1108dee4f76930%7C2d4dad3f50ae47d983a09ae2b1f466f8%7C0%7C0%7C639199948800523337%7CUnknown%7CTWFpbGZsb3d8eyJFbXB0eU1hcGkiOnRydWUsIlYiOiIwLjAuMDAwMCIsIlAiOiJXaW4zMiIsIkFOIjoiTWFpbCIsIldUIjoyfQ%3D%3D%7C0%7C%7C%7C&sdata=2ifmTPlYYhpBwmRDqFMDtciDwuoGUVpQwPoVGYpgeGQ%3D&reserved=0)

**Response**: We thank the editor for the comment. We have modified both figure 4 and 5 according to the PLOS ONE specification.

***6. Please include captions for your Supporting Information files at the end of your manuscript, and update any in-text citations to match accordingly. Please see our Supporting Information guidelines for more information:*** [***http://journals.plos.org/plosone/s/supporting-information***](https://nam04.safelinks.protection.outlook.com/?url=http%3A%2F%2Ftrack.editorialmanager.com%2FCL0%2Fhttp%3A%252F%252Fjournals.plos.org%252Fplosone%252Fs%252Fsupporting-information%2F1%2F010f019f73d9f255-dea6c799-6c0d-4c09-8b66-de605db5cf1a-000000%2F8MOsqXW1xMpkh6HQtPNYwgxoNChe6fapl0CBH9T7l2M%3D258&data=05%7C02%7Cgopal.srivastava%40lsu.edu%7C8a0f4700cc354f0bdd1108dee4f76930%7C2d4dad3f50ae47d983a09ae2b1f466f8%7C0%7C0%7C639199948800547021%7CUnknown%7CTWFpbGZsb3d8eyJFbXB0eU1hcGkiOnRydWUsIlYiOiIwLjAuMDAwMCIsIlAiOiJXaW4zMiIsIkFOIjoiTWFpbCIsIldUIjoyfQ%3D%3D%7C0%7C%7C%7C&sdata=flQt%2BXPM9i%2FULOOiaR07gJAsEN93BGX0MWaIs8ryBNY%3D&reserved=0)***.***

**Response:** We thank you editor for the comment. We have included captions for the supporting information figures and tables at the end of the manuscript, in line with the PLOS ONE submission guidelines.

***7. If the reviewer comments include a recommendation to cite specific previously published works, please review and evaluate these publications to determine whether they are relevant and should be cited. There is no requirement to cite these works unless the editor has indicated otherwise.***

**Response**: Thank you for the comment. Reviewers did not recommend any specific citations.

***Additional Editor Comments: The priority issues that require revisions are as follows:***

***1. The manuscript should provide a more complete description of the machine learning workflow, including the model training procedure, optimizing strategy, hyperparameters, and any modifications made to the selected model. The rationale for choosing YOLOv10 over earlier versions should be also explained, ideally with appropriate comparisons to related approaches.***

**Response**: We thank the editor and reviewers for requesting additional detail on the machine learning workflow. We have revised the manuscript to address each point. We evaluated four optimizers (Adam, AdamW, SGD and NAdam) under matched conditions to identify the best training regime for this task; results are reported in Table 2, with Adam, AdamW, and SGD selected as the final optimizers based on the performance metrices. Optimizers selection procedure is given in **Selecting effective optimizers** and **Selecting epoch and IOU for effective optimizer and model training** sections.

We used YOLOv10b as released by Ultralytics without architectural modification. The contribution of this work is the automated synthetic-annotation pipeline and end-user GUI, not a new detector architecture; we have clarified this scope in the Introduction and Discussion to avoid ambiguity.

A new paragraph in Materials and Methods explains our choice: YOLOv10's NMS-free consistent dual-assignment training removes a documented failure mode of earlier YOLO versions (v5–v9) in which touching or overlapping objects — common in embryo clutches — can be suppressed as duplicate detections, causing systematic under-counting. Head-to-head benchmarks of YOLOv10 against v5, v7, v8, and v9 on standard detection tasks are reported in the original YOLOv10 publication (Wang et al. 2024).

We compared the training performance of the YOLOv10b against RT-DETR and analysis revealed the similar performance of both the methods with Adam and SGD optimizers, but AdamW failed to reach accuracy under 150 epoch. Thus, we went ahead with YOLOv10b for the further analysis and validation. The results for the comparison are mentioned in Benchmark comparison against RT-DETER.

***2. Please ensure that the number of samples used for training, validation, and testing is clearly reported throughout the manuscript. In addition, the rationale for selecting the optimization methods should be described more clearly.***

**Response**: We thank the editor for the comment. We have provided the number of testing and training images in Materials & Method and Results section under *Testing on synthetic and original images* and *Data collection* subsections, respectively. In our approached we cropped images of 200 fertilized embryos, 200 unfertilized eggs, and 100 backgrounds were cropped for the construction of the training set. Training set consisted of a total of 1194 images. To generate an additional testing dataset comprising 120 synthetic images, 50 fertilized embryos, 50 unfertilized eggs, and 10 background samples were extracted from 10 images.

***3. The reviewer 2 raised concerns that the proposed approach is not sufficiently compared with previous method. Please strengthen the discussion by benchmarking your method against relevant existing approaches.***

**Response**: We thank you editor for their suggestion. We compared the training performance of the YOLOv10b against RT-DETR and analysis revealed the similar performance of both the methods with Adam and SGD optimizers, but AdamW failed to reach accuracy under 150 epoch. Thus, we went ahead with YOLOv10b for the further analysis and validation. The results for the comparison are mentioned in Benchmark comparison against RT-DETER.

***4. Please carefully revise the manuscript for formatting consistency and improve the quality of the figures.***

**Response**: We thank editor for their suggestion. We have updated formatting of the manuscript according to the PLOS ONE guidelines and improved figures quality according to the journal specifications.

***Reviewer's Responses to Questions***

***Comments to the Author***

***Have the authors made all data underlying the findings in their manuscript fully available?***

***Review Comments to the Author***

***Reviewer #1: The manuscript presents a YOLOv10b-based tool for automated detection, classification, and quantification of fertilized and unfertilized X. laevis eggs. The use of synthetic data effectively reduces manual annotation efforts, and the model achieved promising performance on both synthetic and experimental datasets. However, several issues should be addressed before the manuscript can be considered for acceptance.***

***1. Manuscript formatting***

***The font size in Lines 115–120 is larger than the rest of the manuscript and should be standardized.***

***Line 131: Please use "Google Pixel 5" instead of "Google Pixel5". Please check and unify the citation style throughout the manuscript. Use the format "Fig. X" instead of "FigX". Equations should be numbered and make referencing in text. The formatting of Table 2 is inconsistent and should be revised.***

**Response:** We thank the reviewer for their valuable comment. We have fixed Google Pixel 5 formatting. The figure numbering has been made in compliance with the journal guidelines which asks that all figures be names Fig X. We have also fixed the Table 2 formatting and now all the statistics shown in the table are rounded up to 3 decimal points.

***2. Dataset description and synthetic data generation. The authors stated that 233 original images were available, but only 53 images were used for training and validation. Please explain why all 233 images were not utilized. After cropping the original images into smaller objects, please report the total number of cropped images, including the numbers of fertilized embryos and unfertilized eggs used for synthetic image generation. The experimental test set contains only 20 images, which appears rather limited and may affect the reliability and generalizability of the results. The authors should present the data in a concise and consistent manner in the tables.***

**Response**: We thank you reviewer for their question. Of the 233 original images provided by NXR, 53 were selected for the synthetic-image generation pipeline based on image-quality criteria: uniform background, in-focus embryos, absence of debris or bubbles, and clear visual separation between fertilized and unfertilized embryos suitable for unambiguous ground-truth labeling. Because our pipeline extracts individual embryos and composites them onto backgrounds to generate training data, the quality of every downstream synthetic image depends on the fidelity of the source crops; including lower-quality source images would propagate labeling noise across the entire synthetic dataset rather than only affecting individual training examples. The remaining 180 images contained conditions (out-of-focus regions, overlapping debris, uneven illumination, or ambiguous developmental stages) that were unsuitable as ground-truth sources but that are representative of real-world imaging conditions the deployed tool must handle — which is why we retained them for the experimental test set rather than the training pool.

The 20 experimental test images were selected from the remaining pool because they contained approximately 300 embryos per dish, yielding a test set of roughly **6,000 individual embryos** on which detection, classification, and counting performance were evaluated. Two considerations motivated this choice. First, reliable evaluation of an automated counter requires reliable manual ground truth: images with ~300 mostly well-separated embryos permit accurate manual annotation by human counters, whereas images with heavy clumping, overlap, or ambiguous developmental stages introduce human-labeling error that would confound the model-accuracy measurement. Second, this density range reflects typical experimental conditions at NXR for the counting task the tool is designed to support. We note that per-object evaluation on ~6,000 embryos provide a substantially larger effective sample size than the image count alone suggests, and the consistency of results across three independently trained optimizer models (Table 3) argues against test-set-specific artifacts. We have added these details and this rationale to Materials and Methods. “The experimental test set consisted of 20 real images from the NXR pool not used in training or synthetic-image generation, selected on the basis of embryo density (approximately 300 embryos per dish) to permit reliable manual ground-truth annotation”

The data collection in materials and method now reflects the reason behind why we chose to use 53 images. The data collection section in the results contains the number of cropped images to create synthetic training, validation and independent synthetic testing set.

***3. Results presentation: The training process is not sufficiently presented. Consider adding training and validation curves (e.g., loss and accuracy) over epochs. Line 334 mentions training YOLOv10b for 50, 100, and 150 epochs, but the corresponding results are not reported. Please provide these results or explain their omission. Precision is exceptionally high (>99%), whereas Recall is considerably lower. The authors should discuss possible reasons for this discrepancy, such as class imbalance, decision thresholds, or biases introduced by synthetic data.***

**Response**: We are grateful to the reviewer for their suggestion. The results for each of the epoch with the best IOU values are given in S1 Table. The reason behind not providing every single combination of the epoch and IOU values was large number of values. We have added all the raw values to the OSF for the reference and mentioned the link in the manuscript. “Detailed list of metrices can be found in <https://osf.io/fm9st> containing all combinations of epoch and IOU values explored as mentioned above.”

We thank the reviewer for identifying this important precision recall pattern in Table 2. We agree the precision-recall asymmetry warrants explicit discussion, and we have added a paragraph to the Discussion. Because the asymmetry is stable across all three optimizers and because both training and validation sets are synthetic in this evaluation, we do not attribute it primarily to optimizer choice or synthetic-to-real domain gap. The most likely contributors are (i) partial embryo-embryo occlusion in our compositing pipeline, which produces irreducibly hidden ground-truth embryos, (ii) sharp confidence distributions from YOLOv10's NMS-free head, which place some true detections just below the reporting threshold and (iii) the mAP@0.5 IoU criterion, which penalizes approximately localized detections as misses. We also note that counting accuracy (94–96% in table 3) remains high despite the recall gap, because missed detections are balanced across classes and do not systematically bias the total count which is the operationally relevant quantity for this tool.

Following added to the manuscript: “Across all three optimizers, precision was consistently near-perfect (0.986–0.997) while recall was lower (0.746–0.803), indicating that the models rarely report false detections but miss a fraction of true embryos. Because this pattern is stable across Adam, AdamW, and SGD, and because both training and validation sets are drawn from the same synthetic generator, we attribute the recall shortfall to factors intrinsic to the pipeline and evaluation rather than to optimizer choice. First, our compositing procedure occasionally produces embryos that partially occlude one another; the underlying embryo remains in the ground-truth annotation but is visually inaccessible, contributing an irreducible floor of false negatives that reduces recall without affecting precision. Second, YOLOv10's NMS-free one-to-one detection head produces sharp confidence distributions, so a subset of true embryos receives scores just below the detection threshold and are counted as misses even though the model has partially learned them. Third, at the mAP@0.5 evaluation criterion, predicted boxes with less than 50% IOU with ground truth are counted as false negatives even when the embryo has been correctly localized in approximate position which could be a source of recall depression for small, densely packed embryos. Class imbalance between fertilized and unfertilized embryos in the training set may contribute additional per-class differences. Importantly, the counting-accuracy metric (Table 3) which is the relevant quantity for a lab tool reaches 94–96% across optimizers, indicating that box-level misses do not translate into large errors in total embryo counts on original images from labs, likely because missed detections are approximately balanced across the two classes.”

***Reviewer #2: The authors have made an attempt to examine the fertilization of the frogs in Africa using ML models.***

***1. How was the data collected is there any lab that using the development of this research.***

**Response**: We thank the reviewer for their comment. The images were collected at the National Xenopus Resource (NXR), Marine Biological Laboratory, MA, USA, 02543, following fertilization of the eggs. NXR collaborated with the Aquatic Germplasm and Genetic Resources Center at Louisiana State University to collect these data in order to explore *X. laevis* egg viability following cryopreservation. This cryopreservation research requires an accurate count of viable versus non-viable embryos after fertilization to determine whether the cryopreservation technique used is effective. Thus, this tool provides researchers with a framework to accurately and quickly determine the viability of eggs after cryopreservation. Data collection procedure can be found in the materials and method under Data collection section.

***2. Why specifically chosen Yolo 10 and why not older version and make comparisons of the related work Is there any changes in the model for obtaining better results.***

**Response**: We thank the reviewer for this comment and have added a paragraph to the Materials and Methods explaining our rationale. Following content was added to the materials ad method section to explain why we chose YOLOv10b was selected for a mechanism-based reason directly relevant to our task: its NMS-free consistent dual-assignment training removes a documented failure mode of earlier YOLO versions (v5–v9), in which touching or overlapping objects which is common in embryo clutches, are suppressed as duplicate detections, causing systematic under-counting. Head-to-head benchmarks of YOLOv10 against v5, v7, v8, and v9 on standard object-detection tasks are reported in the original YOLOv10 paper and consistently favor v10 at matched parameter counts.

We compared the training performance of the YOLOv10b against RT-DETR and analysis revealed the similar performance of both the methods with Adam and SGD optimizers, but AdamW failed to reach accuracy under 150 epoch. Thus, we went ahead with YOLOv10b for the further analysis and validation. The results for the comparison are mentioned in Benchmark comparison against RT-DETER.

***3. Throughout the manuscript mentioned only three optimizers but in the result section Nadam Optimizer results are declared Learning rate value***

**Response**: We appreciate the reviewer for their comment. We agree with the reviewer that we have considered three optimizers for the final analysis and models. Nadam was not considered because Nadam was not able to return performance metrices during the optimizer selection step. We have also added the reason behind not discussing Nadam in downstream analysis in Selecting epoch and IOU for effective optimizer and model training subsection.

***4. All the graphs are not clear it does not have good quality kindly update it.***

**Response**: We thank the reviewer for their comment. All the figures have been updated.

***5. Please include comparison of the proposed with the existing***

**Response**: We thank the reviewer for this comment. We compared the training performance of the YOLOv10b against RT-DETR and analysis revealed the similar performance of both the methods with Adam and SGD optimizers, but AdamW failed to reach accuracy under 150 epoch. Thus, we went ahead with YOLOv10b for the further analysis and validation. The results for the comparison are mentioned in Benchmark comparison against RT-DETER.
